# Review of GPU-based Monte Carlo simulation platforms for transmission and emission tomography in medicine

**DOI:** 10.1088/1361-6560/adfda7

**Published:** 2025-08-29

**Authors:** Yujie Chi, Keith Schubert, Andreu Badal, Emilie Roncali

**Affiliations:** 1 Department of Physics, The University of Texas at Arlington, Arlington, TX, United States of America; 2 School of Engineering & Computer Science, Baylor University, Waco, TX, United States of America; 3 Division of Imaging, Diagnostics, and Software Reliability, U.S. Food and Drug Administration, Silver Spring, MD, United States of America; 4 Departments of Biomedical Engineering and Radiology, University of California, Davis, CA, United States of America

**Keywords:** Monte Carlo, graphic processing unit (GPU), emission tomography, transmission tomography, CUDA, virtual clinical trial, digital twins for healthcare

## Abstract

*Objectives*. Monte Carlo (MC) simulation remains the gold standard for modeling complex physical interactions in transmission and emission tomography, with graphic processing unit (GPU) parallel computing offering unmatched computational performance and enabling practical, large-scale MC applications. In recent years, rapid advancements in both GPU technologies and tomography techniques have been observed. Harnessing emerging GPU capabilities to accelerate MC simulation and strengthen its role in supporting the rapid growth of medical tomography has become an important topic. To provide useful insights, we conducted a comprehensive review of state-of-the-art GPU-accelerated MC simulations in tomography, highlighting current achievements and underdeveloped areas. *Approach*. We reviewed key technical developments across major tomography modalities, including computed tomography (CT), cone-beam CT (CBCT), positron emission tomography (PET), single-photon emission CT, proton CT , emerging techniques, and hybrid modalities. We examined MC simulation methods and major CPU-based MC platforms that have historically supported medical imaging development, followed by a review of GPU acceleration strategies, hardware evolutions, and leading GPU-based MC simulation packages. Future development directions were also discussed. *Main results*. Significant advancements have been achieved in both tomography and MC simulation technologies over the past half-century. The introduction of GPUs has enabled speedups often exceeding 100–1000 times over CPU implementations, providing essential support to the development of new imaging systems. Emerging GPU features like ray-tracing cores, tensor cores, and GPU-execution-friendly transport methods offer further opportunities for performance enhancement. *Significance*. GPU-based MC simulation is expected to remain essential in advancing medical emission and transmission tomography. With the emergence of new concepts such as training machine learning with synthetic data, digital twins for healthcare, and virtual clinical trials, improving hardware portability and modularizing GPU-based MC codes to adapt to these evolving simulation needs represent important directions for future research. This review aims to provide useful insights for researchers, developers, and practitioners in the relevant fields.

## 1. Introduction

Tomography is a biomedical imaging technique that uses penetrating waves or energetic particles and computer technology to form images by sections. The introduction of scintillation detectors to detect radioactive emissions (Cassen *et al* 1951, Anger 1958) led to the first volumetric reconstruction for single photon tomography in 1963 (Kuhl and Edwards 1963). First introduced into medicine in the 1970s (Hounsfield 1973, New *et al* 1974, Ter-Pogossian *et al* 1975), tomography techniques have rapidly advanced through innovations in physics, materials, and computational algorithms, with the goal of enhancing disease detection while minimizing patient harm. Based on the location of imaging sources, state-of-the-art tomography can be generally divided into emission and transmission tomography. Emission tomography quantifies metabolic and physiological activities of living bodies by detecting the internal distributions of radiolabeled tracers, playing an important role in nuclear medicine. Transmission tomography uses external radiation sources to create 3D visualization of anatomical structures, providing essential support in disease diagnosis and treatment.

As innovations in tomography continue, the increasing complexity of systems has created a demand for computational modeling algorithms of growing accuracy. Monte Carlo (MC) simulation (Metropolis and Ulam 1949), which uses stochastic sampling, can reliably solve both deterministic and stochastic problems that are difficult to model with other techniques. By enabling a detailed and precise replication of the underlying physics, MC simulations are particularly effective in investigating new and increasingly complex tomographic system designs. Over the past few decades, a variety of general-purpose and tomography-specific MC simulation packages have been developed. Notable examples include Geant4 (Allison *et al* 2006), EGSnrc (Kawrakow 2000), MCNP (Forster and Godfrey 2006), PENELOPE (Baró *et al* 1995, Salvat 2018), GATE (Jan *et al* 2004), TOPAS (Perl *et al* 2012), among others. These MC simulations have been widely used to advance tomography techniques. However, despite its power, the MC method can be computationally expensive. The statistical error typically scales as $1/\sqrt{N}$, where *N* is the number of simulation histories. For large, complex tomography systems, the time required for highly-precise MC simulations can be extremely long, in the order of days or weeks.

The innovation in graphics processing unit (GPU)-based parallel computing techniques (Owens *et al* 2008) provides a cost-effective solution for MC acceleration. Originally designed for efficient graphics rendering, GPUs provide high data throughput, in contrast to the low-latency nature of CPUs. With the programmability of GPUs greatly boosted by the adoption of the unified shader architecture, using GPUs to solve time-consuming general mathematical problems became feasible (Purcell *et al* 2005, Owens *et al* 2008). The first attempt to develop a GPU-based MC simulation engine for tomography applications was made in 2009, parallelizing photon transport in voxelized geometry and achieving a speed-up factor of 27 with a single GPU compared to a single-core CPU (Badal and Badano 2009). Since then, several GPU-based MC platforms have been developed and widely adopted in tomography simulation studies. For example, gDRR (Jia *et al* 2012a) was developed for simulating cone-beam computed tomography (CBCT) projections, and GGEMS (Bert *et al* 2013) was developed to support both dose and image simulations, amongst others.

In recent years, both the field of tomography and GPU parallel computing have experienced rapid advancements. How to effectively leverage the rapidly increasing GPU computing power to enhance MC simulation performance and expand its versatility to support the growth of the tomography field is an interesting topic. The purpose of this review is to provide a comprehensive overview of state-of-the-art GPU-accelerated MC simulations in tomography, discussing current achievements and highlighting underdeveloped areas, building on previous review efforts by Pratx and Xing (2011), Eklund *et al* (2013), Jia *et al* (2014), Després and Jia (2017). Specific tomography techniques covered include transmission tomography modalities such as CT (Beutel 2000), CBCT (Scarfe *et al* 2006), and proton CT (pCT) (Johnson 2017), as well as emission tomography techniques like positron emission tomography (PET) (Bailey *et al* 2005) and single-photon emission CT (SPECT) (Holly *et al* 2010). Other emerging and hybrid tomography modalities are briefly discussed. We hope this review will serve as a valuable resource for researchers, developers, and practitioners in the field.

## 2. Advances in transmission and emission tomography supported by MC simulations

In both transmission and emission tomography, the mathematical foundation of image reconstruction rests on the assumption that the imaging waves or particles travel in a predictable way between their points of origin and the detection system. However, ray–matter interactions are stochastic and complex, involving ray scattering and the generation of secondary signals. These factors introduce various artifacts into reconstructed images, such as streaks, metal artifacts, signal pseudo-enhancement (Boas *et al* 2012), necessitating robust image reconstruction algorithms that incorporate artifact corrections (Xu *et al* 2015). In parallel, while radiation or particle physics sets intrinsic limits on what can be observed, advancements in emitter and scanner technologies can push the upper limits of achievable image quality. With their substantial capacity to model fundamental interactions precisely, MC simulations play a pivotal role in advancing both reconstruction methods and hardware designs. Additionally, GPU-accelerated MC codes provide unmatched computational performance that enables the use of the MC simulations in practical and large scale applications that are not feasible with legacy general-purpose codes.

In the following subsections, we briefly review the core technological components and historical development of the main transmission and emission tomography modalities, before talking about how GPU-based MC simulations support these innovations.

### 2.1. CT and CBCT

CT and CBCT are the primary x-ray transmission modalities, while the imaging beam spectrum is governed by electron-beam quality, anode target material and design, and the cooling system (Shefer *et al* 2013). Downstream hardware, such as flat metallic filter, bow-tie filter, and collimator, further tailors the beam to enhance image quality and lower patient dose.

To detect the x-ray signal, the scanner consists of two primary functional components: the ray-to-electrical signal converter and the digital readout electronics (Goldman 2007, Shefer *et al* 2013, Hutton *et al* 2018a, Tsoulfanidis and Landsberger 2021). A widely used material for ray absorption is the scintillator crystal that linearly converts x-rays into visible light (Blasse *et al* 1994). This light is then converted into an electric signal by a Silicon photodetector (Anger 1958, Mattson and Vrettos 2002, Shah *et al* 2002, Dolgoshein *et al* 2006). In addition to this two-stage conversion, direct detectors, such as semiconductor-based detectors (Wagenaar 2004), can convert incoming x-ray signals directly into electronic signals. Finally, the digital layer processes the electronic signals into a digital readout for further analysis.

The first CT scan was reported in 1972 (Hounsfield 1973). During the 1970s and 1980s, five successive generations were introduced to reduce the acquisition time, increase the field-of-view, reduce patient dose, and reduce artifacts. Among them, the third generation, with a fan beam and a long, thin, curved detector array, became the foundation for further innovations (Goldman 2007). The sixth and seventh generations emerged in the 1990s and 2000s, introducing slip-ring technology for spiral/helical scanning, with the seventh generation also incorporating a multidetector array. In these CT scanners, the signal from each detector pixel represents an integrated energy deposition from all x-rays arriving at that pixel, which lacks the ability to detect different material compositions. To address this limitation, spectral CT has emerged as a new and promising field by providing tissue-specific, energy-dependent attenuation data (McCollough *et al* 2015, 2020, Greffier *et al* 2023). This includes the development of dual- and multi- energy CT (Johnson 2012, Goo and Goo 2017), and spectrum-sensitive CT scanners, such as layered scanners and photon-counting detectors (Rassouli *et al* 2017, Leng *et al* 2019, Douek *et al* 2023).

CBCT is a natural extension of CT that uses large-area flat panel detectors developed for fluoroscopic imaging instead of dedicated curved detectors (Fahrig *et al* 2021). The flat panel detectors typically use a CsI scintillator coupled with a Silicon photodiode array. The pixel size is usually between 0.1 and 1 mm (and even smaller in dental applications). Due to the small pixel size and continuous panel structure, CBCT scanners do not include septa between the pixels and do not use an external antiscatter grid. CBCT scanners are usually constructed in a portable C-arm configuration instead of a heavy gantry, which results in further physical limitations such as slower scans and increased vibration. Other challenges include incomplete sampling from oblique-angle projections (cone-beam artifacts), increased scatter due to the larger irradiated volume per view, and lower frame rates caused by greater data readout. Even so, CBCT has many practical advantages that make them desirable in numerous clinical applications, such as portability, flexibility in scan trajectory (Hatamikia *et al* 2022), lower cost, and large scanned volume in a single rotation. As a result, improving CBCT image quality has been a continuous focus of research.

### 2.2. PET and SPECT

SPECT and PET are the two main functional medical imaging modalities (Knoll 1983, Raichle 1983, Wernick and Aarsvold 2004, Bailey *et al* 2005, Holly *et al* 2010). SPECT employs single-photon emitters, typically metastable isomers or electron capture nuclei *γ* rays (Pimlott and Sutherland 2011). PET instead uses positron emitters, each of which annihilates with an electron, producing two 511 keV *γ*-rays emitted nearly back-to-back. The detectors used to register these photons are similar to those in x-ray scanners but rely on higher-gain photodetectors such as photomultiplier tubes (PMTs) (Anger 1958), avalanche photodiodes (APDs) (Shah *et al* 2002), or silicon photomultipliers (SiPMs) (Dolgoshein *et al* 2006).

Both PET and SPECT were introduced into medicine in the 1970s. Since then, improving sensitivity and resolution has been the primary focus of technical advancement (Rahmim and Zaidi 2008, Khalil *et al* 2011, Jones and Townsend 2017). Major development include the introduction of new scintillators, semiconductors, APDs, and SiPMs (Peterson and Furenlid 2011, Vaquero and Kinahan 2015, Hutton *et al* 2018a) and new scatter- and attenuation- correction algorithms (Bruyant 2002, Reader and Zaidi 2007). In SPECT imaging, the application of organ-specific collimators and multi-pinhole collimators has boosted sensitivity and image quality (Bruyant 2002, Beekman and Vastenhouw 2004, Beekman and van der Have 2007, Rahmim and Zaidi 2008, Van Audenhaege *et al* 2015). In PET imaging, the introduction of clinical time-of-flight (TOF) PET imaging improves the tracer localization along the line of reaction (Vaquero and Kinahan 2015, Jones and Townsend 2017, Lecoq *et al* 2020). Recently, the use of prompt photons such as Cerenkov photons has shown promise to further detector timing performance. The development of depth-of-interaction (DOI) measurement techniques (Humm *et al* 2003) has enhanced spatial resolution and reduced parallax errors in preclinical PET applications (Cherry *et al* 1997, Vaquero and Kinahan 2015). These innovations have been increasingly supported by MC simulations (Ota *et al* 2018, Lai *et al* 2021).

These innovations have opened the door to new PET and SPECT applications, including preclinical *µ*SPECT and *µ*PET techniques (Cherry *et al* 1997, Chatziioannou 2005, Khalil *et al* 2011), dynamic PET (Samimi *et al* 2020), dual-tracer SPECT (El Fakhri 2012), and immuno-SPECT/PET (Dewulf *et al* 2020, Wei *et al* 2020).

### 2.3. pCT

In charged-particle transmission tomography, such as pCT, the goal is to reconstruct a relative stopping-power map (RSP). To deliver the very low intensities and broad beams needed for imaging, an accelerator paired with a downstream wobbling system is commonly used (Bashkirov *et al* 2016b).

Unlike x-ray CT, where un-attenuated photons travel in straight lines from source to detector, charged particles undergo multiple Coulomb scattering. Each particle’s energy loss and trajectory must therefore be measured individually. Two position-sensitive detector (PSD) arrays, one upstream and one downstream of the body, are used to track the entry and exit coordinates, enabling estimation of the particle’s most-likely path through the patient. Common PSD technologies include scintillating fibers and silicon-strip detectors (Johnson 2017). Residual energy is then recorded with a downstream calorimeter or range detector, typically built from CsI scintillators coupled to PMTs, or from multi-stage hybrids combining both approaches. pCT was first proposed by Cormack in 1963 (Cormack 1963) and explored experimentally in the 1970s and 1980s (Martin *et al* 1975, Cormack and Koehler 1976, Hanson 1979, Kramer *et al* 1980, Hanson *et al* 1982). Over the past two decades, modern prototypes have emerged (Hanson 1979, Benton *et al* 1982, Scaringella *et al* 2013, Saraya *et al* 2014, Johnson *et al* 2015, Naimuddin *et al* 2016, Taylor *et al* 2016, Bashkirov *et al* 2016a), with more under development. These advances have pushed relative RSP precision toward the 1$\%$ target and spatial resolution toward 0.5 mm, making pCT increasingly viable for treatment planning (Schulte *et al* 2005, Johnson 2017). Nonetheless, practical clinical deployment still demands faster detectors to cut scan times (currently 5–10 min) (DeJongh *et al* 2021) and cost-effective solutions such as dedicated, low-cost diagnostic proton accelerators (Martin *et al* 1975, Johnson 2017).

### 2.4. Emerging techniques

Beyond emission and transmission tomography, several other tomography techniques are emerging for medical applications. Among x-ray based techniques, x-ray diffraction/scattering tomography (Harding *et al* 1987, Kleuker *et al* 1998, Jensen *et al* 2011, Geva *et al* 2018) and x-ray phase-contrast tomography (Momose 1995, Bravin *et al* 2012) have been under development. In PET, annihilation from positronium, the intermediate positron–electron bound state, can also be utilized for tomography. The lifetime and annihilation probabilities of positronium are dependent on the surrounding biomolecules, making them useful for monitoring disease progression (Moskal *et al* 2019, 2021). In proton CT, proton-integrating system CT can be used to obtain the average water equivalent path length (Johnson 2017), while scattering proton CT can reconstruct the scattering power of the object (Krah *et al* 2020).

Beyond these modalities, optical tomography using visible or near infrared light to probe tissue absorption and scattering has also emerged as a promising non-invasive imaging approach (Arridge 1999). Optical coherence tomography employs low coherence interferometry has been a routine test choice for ophthalmic diseases and emerging for cardiovascular imaging (Huang *et al* 1991, Wang *et al* 2020). Diffuse optical tomography relies on transillumination measurements to recover 3D maps of tissue absorption and scattering coefficients, enabling functional and molecular imaging in applications such as breast cancer detection and cerebral hemodynamics (Boas *et al* 2001).

Furthermore, ultrasound transmission tomography (UTT) and ultrasound reflection tomography (URT) utilize transmitted and reflected high-frequency ultrasound waves, respectively, to reconstruct acoustic attenuation, sound-speed maps, and pulse–echo backscatter distributions (Greenleaf *et al* 1974, 1975, Kak 1979, Jago and Whittingham 1991, Gemmeke and Ruiter 2007). Both UTT and URT have shown promise in applications such as breast cancer detection.

### 2.5. Hybrid modalities

Alongside technical advancements in each of these tomography modalities, a key direction in instrument development is hybrid imaging, also known as multi-modality imaging (Patton *et al* 2009, Martí-Bonmatí *et al* 2010). Representative techniques for hybrid diagnostic imaging include PET/CT (Townsend *et al* 2004, von Schulthess *et al* 2006), SPECT/CT (Buck *et al* 2008, Mariani *et al* 2010), PET/MRI (Judenhofer *et al* 2008, Ehman *et al* 2017), SPECT/MRI (Hutton *et al* 2018b), ultrasound/CT (Gemmeke and Ruiter 2007, Kratkiewicz *et al* 2022), and photoacoustic tomography (Wang and Hu 2012), among others. These hybrid imaging modalities provide complementary structural-functional or functional–functional imaging information with co-registration, enhancing both image quality and disease diagnosis. Furthermore, the integration of imaging modalities with therapeutic modalities, such as CBCT/linac (Srinivasan *et al* 2014), PET/linac (Oderinde *et al* 2021, Shirvani *et al* 2021), and in-beam PET (Parodi *et al* 2001), enables image-guided radiotherapy, combining structural and biological insights for more precise cancer treatment. Challenges to be addressed include ensuring modality compatibility, maximizing the hybrid effect, and minimizing the potential for increased harm to the body (Patton *et al* 2009).

All these techniques continue to undergo significant development, with MC techniques playing a critical role in advancing them through deeper understanding of the underlying physics, system design, and image processing optimization. MC simulations are described in the next section.

## 3. MC simulation in transmission and emission tomography

### 3.1. General working principles

Simulation models are approximations of real-world systems. Therefore, it is important to balance between approximation accuracy and model complexity. In MC applications for tomography, the following principles are typically utilized in balancing accuracy and complexity.

First, material composition and geometry of the medium needs a proper discretization. For biological bodies, a common practice is to uniformly discretize the body into small voxels. For beam source and detector simulations, parametrized geometries are typically used to describe layered materials with fine structures (Rogers *et al* 1995, Sulkimo and Vuoskoski 1996). Voxelized geometry is typically particle navigation friendly, while it can be memory-unfriendly when describing a large volume with a fine resolution. On the other hand, transporting the particle from one layer to another can be computationally more expensive in a parametrized geometry, yet it can be memory efficient in geometry description. An alternative strategy employs triangle-mesh ray tracing with a more versatile geometry representation (Badal *et al* 2009). Its limitations can be large memory occupation and complex particle navigation. Nonetheless, in all these geometry modelings, boundary crossing effect needs to be considered carefully when sampling particle step sizes, especially with heterogeneous boundaries (Rogers *et al* 1990). Techniques such as the Woodcock trick have been developed to effectively address frequent boundary checking problems (Woodcock *et al* 1965).

Second, ray transport and interactions with the medium need to be discretized in both space and time (Rubinstein and Kroese 2016). Although the interaction cross section (i.e. probability) can be measured or theoretically computed, the distance a ray travels before interacting with the medium is a stochastic process and cannot be directly determined from the macroscopic properties of the medium. Instead, a discretization model allows us to sample ray travel and interactions, with the net effect following the cross-section measurements. In x-ray and gamma-ray transport, rays are modeled as photon particles, and the entire track is discretized into interaction points, connected by straight, free-flying segments (Rogers *et al* 1990). For electrons with energy in the medical imaging range, the most commonly used model is the class I and II condensed history approximation, developed based on multiple scattering theory. In this approach, a large number of ‘soft’ electron transport events and collisions are combined into a single step, and ‘hard’ interactions are simulated individually (Berger 1963, Rogers *et al* 1990, Kawrakow 2000). In proton transport, in addition to using a similar strategy for interactions with on-shell electrons, another consideration is the proton’s nuclear elastic and inelastic interactions. These interactions can become extremely complex, especially when accounting for fragmentation and secondary particle production (Dedes *et al* 2022).

Third, physical interactions must be properly approximated. When primary rays interact with a medium, they can undergo various interactions and generate secondary effects. The selected physics model should accurately reflect relevant interactions within the simulation scope, while processes with minimal impact can be reasonably omitted to preserve computational efficiency. For example, in the energy range relevant to medical imaging, the primary inelastic interactions for x-rays and gamma-rays are Compton scattering and the photoelectric effect. These processes may lead to secondary electron production, the Auger effect, and secondary characteristic x-ray emission. However, if the simulation focuses on tracking the primary ray, these secondary processes can be usually ignored. Conversely, in simulations focused on imaging dose or detailed detector behavior, some of these secondary processes need to be tracked in greater detail, while the need to increase the level of detail depends on geometry, energy, material, among other factors.

Meanwhile, due to the inherent randomness of the MC method, simulation results may have significant variance. Various variance reduction methods, such as importance sampling, have been investigated to reduce variance without sacrificing efficiency (Niederreiter 1992, Kleijnen *et al* 2010, García-Pareja *et al* 2021). Non-analog MC, which introduce biases to the simulation, can potentially improve efficiency by focusing on important regions compared to analog MC methods (Brown and Martin 1984). Additionally, quasi-random sampling, with low discrepancy, can be more efficient than pseudo-random sampling in achieving MC simulation convergence (Niederreiter 1992, Lin *et al* 2021).

Numerous MC packages have been developed for broad use or more specific research purposes. The next section summarizes available packages, whose implementation on GPU is later discussed in this review.

### 3.2. Major MC simulation packages

In transmission and emission tomography, commonly used MC simulation packages can be categorized into general-purpose packages and modality- or function-specific packages. General-purpose packages are typically highly flexible, capable of simulating a broad spectrum of particles across a wide energy range, with rich cross-section models and geometry configurations. However, they can be complex for beginners, especially for those not proficient in programming or physics interaction details. On the other hand, dedicated packages are tailored and optimized for specific simulation tasks. While they are generally easier to use, they may lack flexibility if the user wishes to modify the simulation scope. Below, we briefly summarize the widely used state-of-the-art general-purpose and dedicated MC packages in transmission and emission tomography, with a particular emphasis on their functional coverage of the relevant modalities.

ETRAN (Electron TRANsport) (Seltzer 1988b, 1991) is one of the early codes developed to simulate coupled electron–photon transport in the energy range of 1 keV to 100 GeV. It was FORTRAN-based, with the first version released in 1968 (Berger 1968). This code introduced the condensed history approximation for electron transport (Berger 1963), which laid the foundation for electron transport modeling in all subsequent MC codes above the keV range. However, while ETRAN emphasizes accurate physical interaction modeling, its limited geometric support, restricted to slabs or cylinders with a few materials (Seltzer 1988a), constrains its applicability in medical tomography simulations. Nonetheless, it has served as the physics engine for codes with more versatile geometrical modeling, such as integrated tiger series (Halbleib and Mehlhorn 1986).

MCNP (MC code for Neutron and Photon transport) (Forster and Godfrey 2006, Sood 2017, Kulesza *et al* 2022b) is a FORTRAN-based general-purpose MC code, first released by Los Alamos National Laboratory in 1977 after decades of development on special-purpose MC codes for neutron and photon transport (Forster and Godfrey 2006). At this stage, MCNP has released version 6.3, which supports the tracking of 37 particle types across a broad energy range, including the transport and detection of photons, electrons, and protons for both emission and transmission tomography. Since version 6.3, MCNP has adopted a mixed FORTRAN/C/C++ programming paradigm (Kulesza *et al* 2022a).

EGS (Electron Gamma Shower) is a general-purpose MC code developed by the Stanford Linear Accelerator Center to simulate coupled electron–photon transport in various materials and geometries (Hirayama *et al* 2005). It was developed in both FORTRAN and MORTRAN, with some versions using standard FORTRAN and others employing MORTRAN macros. Upon the release of EGS version 4, researchers from the National Research Council of Canada introduced EGSnrc, which brought significant improvements in the accuracy of low-keV electron transport (Kawrakow 2000). EGSnrc was written in MORTRAN and has provided a C interface since version 4. Additionally, years of development of the EGS series enhanced its capability to simulate complex photon interactions, including phenomena such as fluorescent radiation, Doppler broadening, and more (Hirayama *et al* 2005). These advancements made EGSnrc particularly useful for medical physics applications (Kawrakow and Rogers 2000). Additionally, the BEAM/BEAMnrc codes were developed based on the EGS framework, with a specific focus on simulating radiotherapy beam sources, which also proves useful for simulating radiation imaging sources (Rogers *et al* 1995, 2009).

PENELOPE (PENetration and Energy LOss of Positrons and Electrons) (Baró *et al* 1995, Salvat 2018) is an open-source FORTRAN-based simulation package designed to support coupled electron–photon transport in arbitrary materials across a wide energy range, from a few hundred eV to about 1 GeV. The code system includes detailed simulation of hard events while, optionally, soft interactions can be treated using a condensed history approach. PENELOPE has been extended to support the transport of protons and other heavy charged particles (Salvat 2013). PENELOPE also features versatile support for quadratic geometry through its geometry package, PENGEOM, allowing for flexible particle navigation in complex geometries (Almansa *et al* 2025). Its geometrical model has been further extended to support voxelized geometries (Sempau *et al* 2011) and CAD-derived triangle-mesh representations of free-form objects (Borglund *et al* 2004, Badal *et al* 2009). PENELOPE has been recently translated into C++ and combined with GEANT4 (Asai *et al* 2021).

Geant4 (GEometry ANd Tracking, version 4) (Agostinelli *et al* 2003, Allison *et al* 2016) is a C++-based object-oriented simulation toolkit developed through worldwide collaborative efforts, designed to support the transport of a wide range of particles through complex geometries across an extensive energy range. It features a built-in geometry module and accepts external voxelized geometries in multiple formats, including standard medical files, as well as boundary-representation models from CAD systems (Sulkimo and Vuoskoski 1996). It supports the simulation of a variety of physics processes, such as electromagnetic, hadronic, and optical interactions, making it a powerful tool for applications in medical image simulations (Allison *et al* 2006). In 2006, a Geant4-Python interface was introduced (Murakami and Yoshida 2006). Building upon the Geant4 framework, the OpenGATE collaboration developed GATE (the Geant4 Application for Tomographic Emission) (Jan *et al* 2004), which is specifically dedicated to simulating PET and SPECT imaging modalities, including in-beam PET. Additionally, TOPAS (TOol for PArticle Simulation) (Perl *et al* 2012) was released to facilitate proton therapy simulations, offering a specialized toolset for accurate radiation transport and dosimetry in proton clinical settings.

FLUKA (Fluktuierende Kaskade) (Fasso *et al* 1995, Ferrari *et al* 2005) is another FORTRAN-based general purpose MC code for particle transport co-developed by the European Organization for Nuclear Research and the Italian Institute for Nuclear Physics . It supports the transport of more than 60 types of particles, including photon, neutron, charged particles, and optical photons in a broad energy range. Both analog and biased transport modes are available. It also supports both voxelized geometries and complex parameterized geometries through its Combinatorial Geometry package.

Dose planning method (DPM) (Sempau *et al* 2000) is a PENELOPE-based MC code optimized for electron and photon radiotherapy dose computation. It supports large step size-based electron and photon transport (i.e. jumping over multiple voxels in a single step) using delta scattering, without compromising dosimetric accuracy, making it run much faster than CPU-based general MC codes.

In ultrasound tomography, the absorption and scattering of ultrasound by structures smaller than the wavelength, as well as reflection and refraction by larger structures, need to be modeled. While dominant approaches typically rely on deterministic methods for both processes, recent studies have introduced stochastic MC methods (Mattausch *et al* 2018, Li *et al* 2021, Amadou *et al* 2024, Duelmer *et al* 2025), resulting in significantly more realistic simulations of ultrasound interactions.

In optical tomography, numerous MC codes have been developed to model optical photon transport in biological tissues (Arridge and Schotland 2009, Zhu and Liu 2013, Periyasamy and Pramanik 2017). The first few codes, developed in the 1980s, focused on light propagation in relatively simple tissue geometries (Wilson and Adam 1983, Flock *et al* 1989, Prahl 1989). MCML (MC Modeling of Light transport in multilayered tissues), released in 1995, introduced multi-computer-system portability (Wang *et al* 1995). Most recent developments have expanded the modeling capabilities to include more complex and realistic tissue geometries (Boas *et al* 2002, Fang 2010, Shen and Wang 2010, Periyasamy and Pramanik 2014).

## 4. General purpose-graphical processing unit (GPGPU) based MC simulation in transmission and emission tomography

Alongside the rich development of CPU-based MC simulation packages that have provided versatile support for tomography research, their low computational efficiency has significantly limited broader applications in large-scale practical scenarios. Understanding the root causes of this efficiency gap requires examining how MC codes are executed at the hardware level. Regardless of the high-level programming language used (e.g. C, FORTRAN, or Python), MC codes are ultimately translated into a series of binary instructions when executed on computing hardware. These instructions can be broadly categorized into branching instructions, memory instructions, and arithmetic instructions, making these three operations the primary determinants of code execution efficiency. At the machine code level, performance can be characterized by three fundamental metrics: latency, throughput, and concurrency.

**Figure 1.**
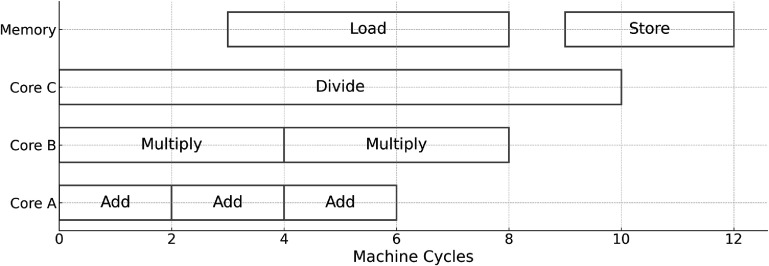
The illustration of a mixed instruction schedule spanning multiple machine cycles. Instruction latency ranges from 1 to 10 cycles, concurrency varies from 1 to 4 instructions at a given time, and the total throughput is 8 instructions completed over 12 cycles.

In practical MC simulation scenarios, diverse arithmetic and memory operations are combined (as illustrated in figure 1), making the consideration of average performance metrics particularly meaningful. The fundamental relationship between these average metrics is encapsulated by Little’s Law (Little 2011), which can be formulated as:

\begin{equation*} \mathrm{instruction \ throughput} = \frac{\mathrm{mean\ concurrent \ instruction \ execution}}{\mathrm{mean\ instruction \ latency}}.\end{equation*}

**Figure 2.**
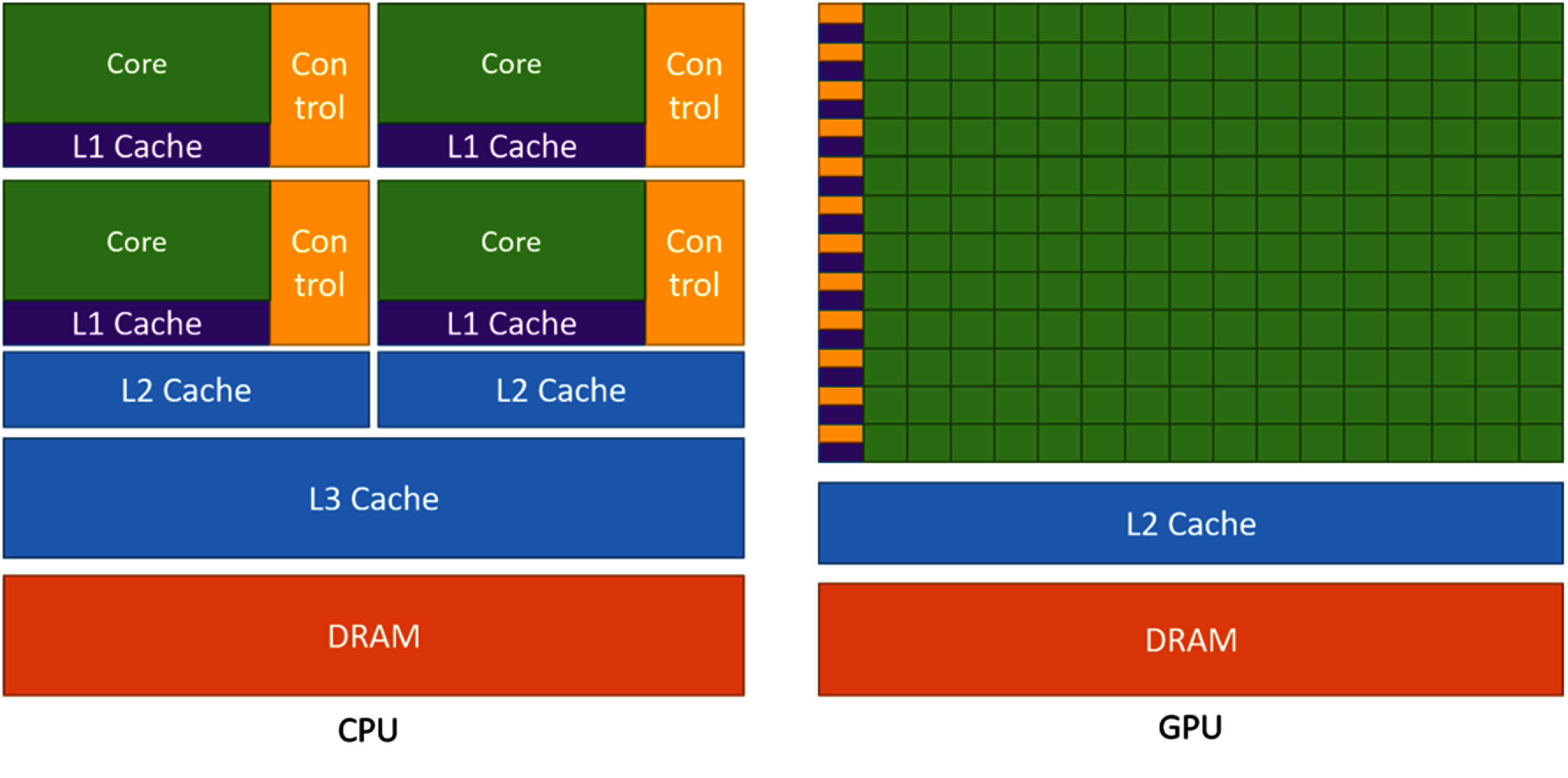
CPU vs. GPU Architecture: The CPU (left) has a few powerful cores with a deep cache hierarchy and dedicated control unit per core, optimizing for low-latency tasks. The GPU (right) features many smaller cores with a flatter memory structure, maximizing parallel processing efficiency. (*Source*: NVIDIA https://docs.nvidia.com/cuda/cuda-c-programming-guide/index.html). Reproduced with permission from NVIDIA Corporation

This equation underscores two main strategies to enhance throughput: either by increasing concurrency, thereby enabling the execution of more simultaneous instructions, or by reducing latency, thereby speeding up individual instruction processing. CPUs and GPUs utilize different strategies in architecture designs for optimal computational performance (illustrated in figure 2).

CPUs prioritize low-latency execution. As shown in the left part of figure 2, they feature an efficient memory system, including an hierarchical cache structure. Typical CPU latencies range from 1–4 cycles for arithmetic operations to tens of cycles for memory access, depending on cache efficiency. This design makes CPUs well-suited for sequential processing, complex decision-making, and control-heavy tasks. In contrast, GPUs maximize concurrency and throughput via massively parallel computing architectures, as illustrated in the right part of figure 2. The streaming multiprocessor (SM) (a term used by NVIDIA and AMD GPUs) serves as the core computational unit, housing numerous stream processors (SPs) that execute thousands of threads in parallel. High arithmetic throughput is then achieved through single instruction multiple threads (SIMT) execution, latency-hiding techniques, and high-bandwidth memory retrieval. However, it is important to note that GPUs rely on CPUs for task coordination. CPU-GPU data transfers via PCIe can become performance bottlenecks if not properly managed.

To examine the role of GPUs in accelerating MC simulations for transmission and emission tomography, the following sections will first overview GPU architectures and programming techniques, followed by a review of acceleration strategies, major GPU-based MC packages, and their applications in the tomography field.

### 4.1. GPGPU platforms and programming

Around 2000, Mark Harris (Harris 2005) coined the term GPGPU to describe the application of GPUs in non-graphics computations. Today, the GPGPU market is dominated by NVIDIA, AMD, and Intel, with NVIDIA being the most prominent player. In parallel, several programming platforms to support GPU computing have been developed. Dedicated options include NVIDIA’s CUDA, introduced in 2006 (Sanders and Kandrot 2010), and AMD’s ROCm, launched in 2016 (Otterness and Anderson 2020). Cross-platform solutions contain OpenCL from the Khronos Group (Munshi 2009) and Microsoft’s DirectCompute (Ni 2009), among others.

**Figure 3.**
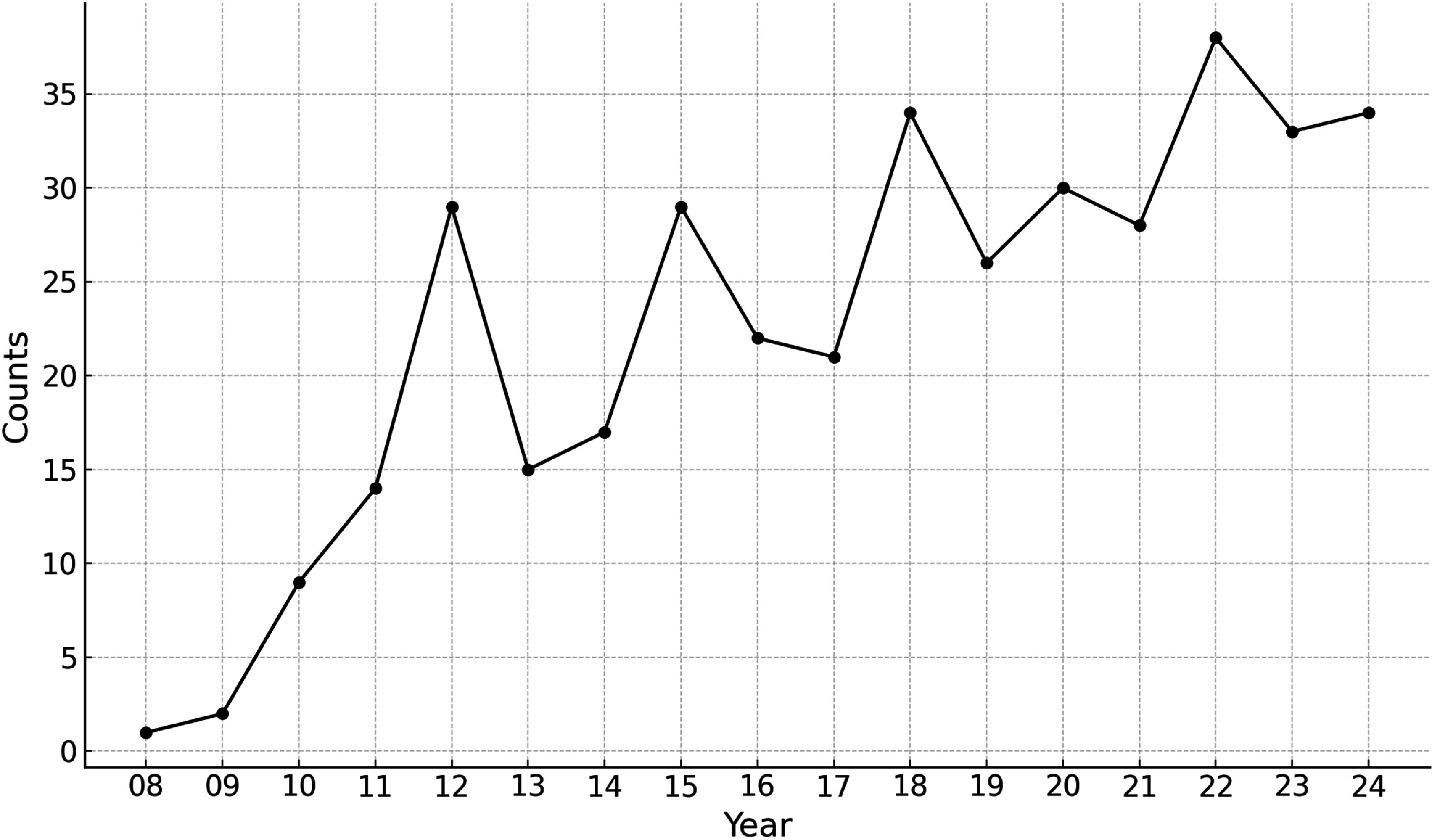
PubMed Publications per year from 2008 to 2024 with keywords ‘GPU Monte Carlo’.

A metasearch of the PubMed database using the keywords ‘GPU Monte Carlo’ revealed a steadily increasing trend in publications from 2008 to 2024, as shown in figure 3. Further analysis using the keywords ‘Monte Carlo’ combined with GPU computing platform names in titles or abstracts shows that CUDA overwhelmingly dominates GPU-based MC applications in the medical field, with 50 publications referencing CUDA compared to only 11 referencing OpenCL. Thus, in this review paper, we specifically address the evolution of NVIDIA GPUs and the corresponding CUDA programming framework. For readers interested in other GPU platforms, we hope it still provides valuable insights as they share fundamental principles of parallel computing. For detailed application instructions on other platforms, readers are encouraged to refer to relevant programming tutorials or documentation.

In table 1, we summarize major NVIDIA GPU microarchitectures, tracing their evolution from Tesla (compute capability 2.0, 2006) to Hopper (compute capability 9.0, 2022). This progression reflects advancements in both arithmetic logic units and memory architecture. Across generations, the number of SMs per GPU has steadily increased, alongside continuous optimization of the types and counts of functional cores within each SM. Recent architectures have integrated tensor cores for ultra-fast mixed-precision matrix operations. These advancements have led to a substantial rise in peak arithmetic throughput. For instance, peak single-precision (FP32) performance rose from roughly 1 TFLOPS on GF110 to around 60 TFLOPS on GH100. Memory management has also advanced, with larger on-chip memory per SM and transitions from GDDR5 to HBM2 and HBM3 DRAM, boosting memory bandwidth from 60 Gbps on GK104 to 3 Tbps on GH100.

**Table 1.** Evolution of NVIDIA GPU Architectures and CUDA Versions.

	Fermi	Kepler	Maxwell	Pascal	Volta	Turing	Ampere	Hopper
Tesla GPU	GF100	GK104	GM200	GP100	GV100	TU104	GA100	GH100
Compute capability	2.0	3.0	5.3	6.0	7.0	7.5	8.0	9.0

Arithmetic logical unit techniques								

SM	16	8	24	56	84	72	128	144
FP32 Cores/SM	32	192	128	64	64	64	64	128
FP64 Cores/SM	—	64	4	32	32	2	32	64
Tensor Cores/SM	N	N	N	N	8	8	4	4

Memory management techniques								

Max register size/SM	64KB	256KB	256KB	256KB	256KB	256KB	256KB	256KB
Shared memory/SM	48KB^a^	48KB^a^	96KB	64KB	96KB^a^	96KB^a^	164KB^a^	228KB^a^
DRAM	GDDR5	GDDR5	GDDR5	HBM2	HBM2	HBM2	HBM2	HBM3^b^

CUDA techniques								

CUDA version	3.0–8.0	5.0–10.2	6.5–12.8	8.0–12.8	9.0–12.8	10–12.8	11–12.8	11.8–12.8
Threads/Warp	32	32	32	32	32	32	32	32
Warps/SM	48	64	64	64	64	64	64	64
Threads/SM	1536	2048	2048	2048	2048	2048	2048	2048
Blocks/SM	8	16	32	32	32	32	32	32
Threads/Block	1024	1024	1024	1024	1024	1024	1024	1024
Thread block cluster	N	N	N	N	N	N	N	Y
Registers/Thread	63	63	255	255	255	255	255	255

Advanced CPU-to-GPU, GPU-to-GPU communication techniques								

Hyper-Q	N	N	Y	Y	Y	Y	Y	Y
Dynamic parallelism	N	N	Y	Y	Y	Y	Y	Y
NV-Link	N	N	N	Y	Y	Y	Y	Y
Unified memory	N	N	N	Y	Y	Y	Y	Y

*Note:* — means not specified.

^a^ Maximally configurable shared memory size in combined shared memory/data cache architecture.

^b^ In some versions of GH100, HBM2e rather than HBM3 is used.

In parallel, CUDA software has also advanced from version 1.0–12.3. Table 1 shows the supported CUDA versions for each compute capability. Compatibility between CUDA device code and compute capability is achievable at both assembly level and binary level using appropriate compiler flags. The CUDA programming model follows a hierarchical execution structure, where computations are organized into kernels that launch threads, thread blocks, and grids. At the hardware level, SIMT execution remains the foundation, with warp scheduling used to hide memory and instruction latencies. Efficient thread synchronization at different levels, such as warps, thread blocks, and grids, are managed by the hierarchical on-chip cache and shared memory. Since GV100, per-warp scheduling has been refined to sub-warp granularity, allowing better control over thread divergence and reconvergence. The Hopper GH100 further extends the CUDA programming model with the Thread Block Cluster architecture, improving thread cooperation across SMs.

Additionally, NVIDIA GPUs have continuously improved CPU-to-GPU and GPU-to-GPU communication techniques, introducing Hyper-Q, Dynamic Parallelism, NV-Link, and Unified Memory, among others. These advancements, combined with the continued evolution of CUDA and the CUDA Toolkit, enhance GPU programmability for complex computing tasks, making it more accessible even to less experienced developers. Interested readers can refer to NVIDIA’s white papers for detailed highlights on GPU architecture across different compute capabilities and consult the ‘CUDA C++ Programming Guide’ for in-depth information on CUDA programming.

### 4.2. GPGPU acceleration techniques for MC simulation

**Figure 4.**
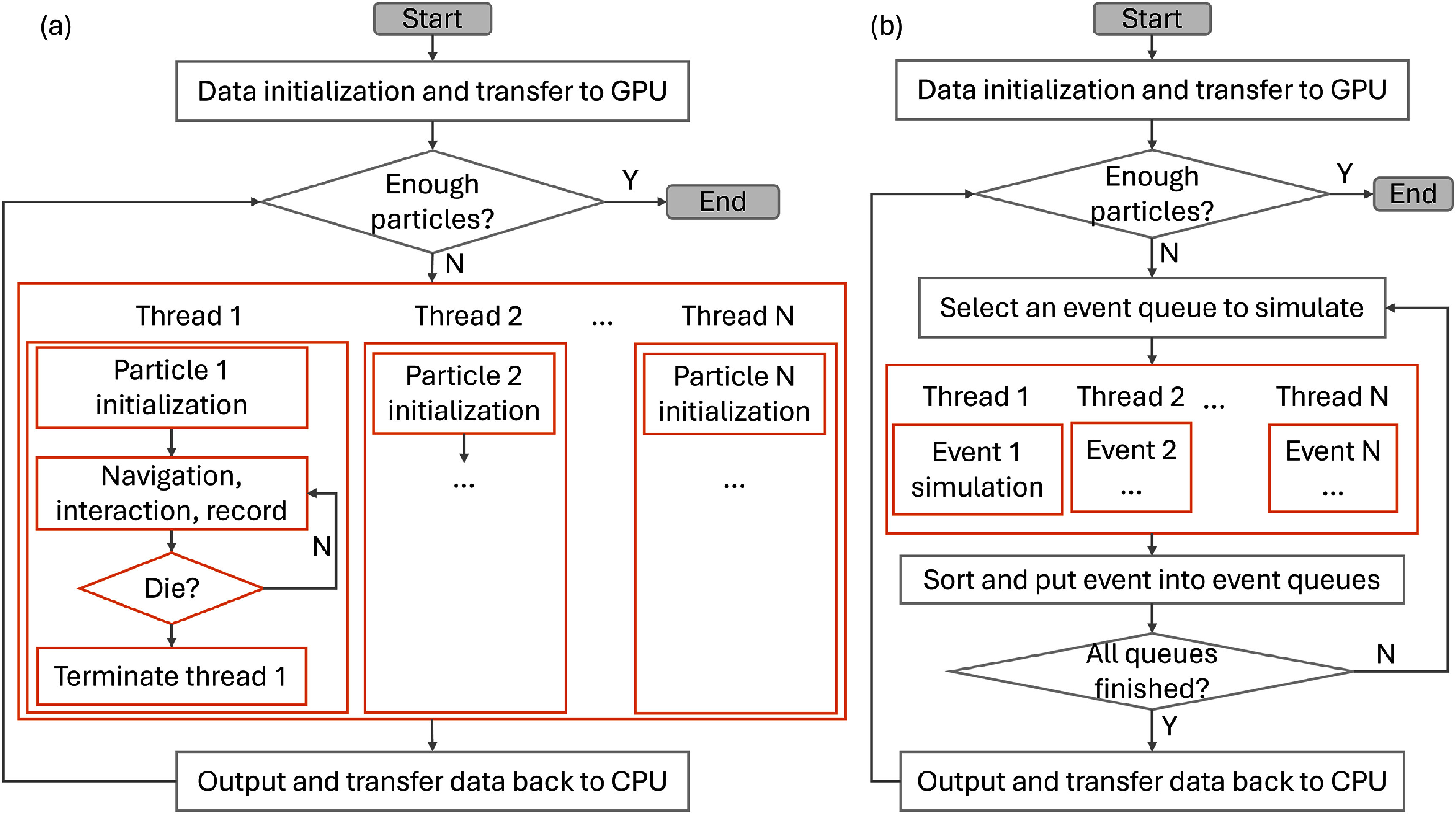
An illustration of GPU-based MC simulation for particle transport (single particle type) using (a) a history-based strategy and (b) an event-based approach. Orange blocks indicate GPU kernel executions.

When applying GPGPU to accelerate MC simulations, it is often natural to adopt history-based transport (Brown and Martin 1984), where each thread simulates a single particle history from birth to death considering independence among histories, as illustrated in figure 4(a). In contrast, event-based transport organizes GPU kernels by event type, such that each particle history is processed across multiple kernels throughout its lifetime (Brown and Martin 1984), as illustrated in figure 4(b). In both approaches, however, a simple and direct transition from CPU to GPU implementations may not yield optimal performance. For instance, Jia *et al* showed that an initial GPU implementation of the DPM algorithm, based on history-based transport, achieved only a six-fold speedup (Jia *et al* 2010), while a carefully optimized version reached nearly 87-fold acceleration (Jia *et al* 2011).

Insight into optimizing computational performance can be drawn from equation (1), which indicates that achieving peak instruction throughput requires maximizing instruction-level concurrency and minimizing latency. For GPU acceleration, this principle translates into careful management of latency hiding, SIMT execution model, memory bandwidth and latency, and arithmetic throughput.

Effective latency hiding demands both sufficient threads per block and enough thread blocks to maintain a high level of concurrent warp scheduling. Typically, the total number of threads per kernel should significantly exceed the thread limits of the GPU. However, this strategy must be balanced with per-thread memory usage, such as registers and shared memory. Hamilton and Evans (2019) demonstrated that, in a history-based implementation of continuous-energy neutron MC transport, latency hiding was substantially constrained by high register usage per thread. In their study, even a low occupancy of approximately 15% was enough to fully saturate GPU resources. In contrast, event-based transport can support much higher occupancy levels before reaching saturation, due to its lower register usage per thread. Therefore, maximizing latency hiding requires careful management and optimization of per-thread memory consumption.

Under the SIMT execution model, it is desirable for all 32 threads within a warp to execute the same instruction simultaneously. If thread divergence occurs, execution within the warp becomes serialized, significantly reducing thread-level concurrency and computational efficiency. This issue is particularly significant in history-based transport, where diverse particle energies, particle types, and interaction cross-sections frequently lead to thread divergence. To address this, both GPUMCD (Hissoiny *et al* 2011a) and gDPM 2.0 (Jia *et al* 2011) employed a strategy of separating photon transport and secondary electron transport into distinct GPU kernels in coupled photon–electron MC simulations, significantly reducing divergence. gDPM 2.0 also grouped particles with similar particle energies to further reduce thread divergence. GPUMCD additionally incorporated stream compaction (Billeter *et al* 2009) and persistent threads with particle pooling to reduce thread divergence in electron transport.

Achieving peak memory bandwidth requires coalesced data access, while minimizing memory latency necessitates careful utilization of GPU memory hierarchies. By organizing cross-section, geometry, particle, and other relevant data into suitable formats and memory locations, both memory throughput and latency can be effectively optimized. For example, particle coordinates and energies can be structured using CUDA’s built-in vector format (float4), automatically satisfying data alignment requirements for efficient global memory access. Jia *et al* (2011) demonstrated a notable performance improvement by utilizing GPU texture memory to perform linear interpolation on cross-section data. Additionally, Chi *et al* (2016) employed shared memory to optimize parameterized geometry navigation, enhancing particle transport efficiency.

Maximizing arithmetic throughput involves implementing high-throughput arithmetic operations and leveraging CUDA’s built-in functions. In MC simulations, it is common to use single-precision floating-point arithmetic instead of double-precision to significantly improve arithmetic throughput. Additionally, applying CUDA’s built-in libraries, such as cuRAND for random number generation in MC simulation, often outperform custom implementations in efficiency (Hissoiny *et al* 2011a). Furthermore, the concept of arithmetic intensity, that is the ratio of arithmetic operations to the amount of data accessed from memory, can be used to determine whether the kernel is compute-bound or memory-bound, guiding further performance optimizations.

Except for utilizing CUDA cores for MC acceleration, recent efforts have highlighted the potential of using ray tracing (RT) cores and tensor cores for further performance gains. Since the introduction of NVIDIA’s Turing architecture, RT cores have been integrated into GPUs to enable hardware-level acceleration of ray-tracing operations. Notably, several research groups have investigated the use of RT cores to accelerate MC simulations beyond traditional CUDA-based approaches (Salmon and McIntosh-Smith 2019, Cui *et al* 2024, Lee and Ye 2024). The core idea is to reformulate geometric operations involved in particle navigation as ray-tracing problems, utilizing triangle mesh representations and bounding volume hierarchy tree structures. Salmon and McIntosh-Smith (2019) were among the first to explore the application of RT cores for accelerating MC codes, while Lee and Ye (2024) brought the technique to explicitly accelerate MC codes for medical applications.

### 4.3. Major GPGPU-based MC simulation packages

We summarize the main GPGPU-based MC simulation packages developed for transmission and emission tomography applications in table 2, broadly categorizing them into dose computation and imaging simulation engines. Below, we briefly discuss each package.

**Table 2.** GPU-based Monte Carlo simulation packages for transmission and emission tomography.

GPU Package	Year	Algorithm	Particle	Geometry	Transport	Function	Speedup Factor^a^	References
Dose computation engines in diagnostic and therapeutic energy range								

gDPM	2010	DPM	$\gamma, e^-$	Voxelized, Parametrized	History	Dose	$\sim 87\times$	Jia *et al* (2010, 2011), Chi *et al* (2016)
GPUMCD	2011	*γ*: analog; $e^-$: Class II	$\gamma, e^-$	Voxelized	History	Dose	$\sim 200\times$	Hissoiny *et al* (2011a, 2011b)
GMC	2012	Geant4	$\gamma, e^-$	Voxelized	Event	Dose	$\sim 4860\times$	Jahnke *et al* (2012)
gCTD	2012		*γ*	Voxelized	History	Dose	$\sim 400\times$	Jia *et al* (2012c)
gPMC	2012	Class II	p	Voxelized	History	Dose	2-orders	Jia *et al* (2012a)
GGEMS	2013	Geant4	$\gamma, e^-$	Voxelized	History	Dose	$\sim 90\times$	Bert *et al* (2013), Lemaréchal *et al* (2015)
goCMC	2017	Class II	C ion	Voxelized	History	Dose	2-orders	Qin *et al* (2017)
pGPUMCD	2019	Class II	p	Voxelized	History	Dose	2-orders	Maneval *et al* (2019)
RT2	2024	$\gamma, e^\pm$: EGSnrc n: group-wise	$\gamma, e^\pm$, n	Mesh	Event	General	$\sim 300\times$	Lee and Ye (2024)

Emission and transmission image simulation engines								

MCGPU	2009	PENELOPE	*γ*	Voxelized	History	General	$\sim 27\times$	Badal and Badano (2009), Badal *et al* (2021)
CUDA EGS	2011	EGS	*γ*	Voxelized	History	General	$\sim 40\times$	Lippuner and Elbakri (2011)
gDRR	2012		*γ*	Voxelized	History	CBCT		Jia *et al* (2012b)
GGEMS	2013	Geant4	$\gamma, e^-$	Voxelized	History	General	$\sim 800\times$	Bert *et al* (2013)
GGEMS-SPECT	2016	GGEMS	$\gamma, e^-$	Voxelized, analytical	History	SPECT	$\sim 71\times$	Garcia *et al* (2016)
GGEMS-PET	2019	GGEMS	*γ*	Voxelized	History	PET	$\sim 776\times$	Ma *et al* (2019)
gPET	2019	gDPM	$e^+$, *γ*	Voxelized, Parametrized	History	PET	$\sim 500\times$	Lai *et al* (2019)
MCGPU-PET	2024	PENELOPE	*γ*	Voxelized	History	PET	3-orders	Herraiz *et al* (2024)
UMC-PET	2024	PENELOPE	$e^+$, *γ*	Voxelized	History	PET	$\sim 500\times$	Galve *et al* (2024)

*Note:* ^a^ Speedup factors are as reported by each package and may depend on GPU/CPU architecture, the reference CPU-based MC code, the physics covered, and the specific test cases. Direct comparison across packages may be misleading.

#### 4.3.1. Dose computation engines

DPM-based gDPM, which performs dose calculations through history-based photon–electron transport in voxelized geometry, was initially developed in 2010 (Jia *et al* 2010), achieving a speedup up to six times. The performance was improved to 87-fold in 2011 (Jia *et al* 2011) by managing thread divergence and memory latency. In 2016, parameterized geometry features were introduced (Chi *et al* 2016). The same group then developed gCTD in 2012 for CT/CBCT imaging dose computation (Jia *et al* 2012c), gPMC in 2012 for proton dose computation (Jia *et al* 2012a), and goCMC in 2017 for carbon ion dose calculation (Qin *et al* 2017). Notably, goCMC was built using the OpenCL architecture.

GPUMCD (Hissoiny *et al* 2011a) is another history-based photon–electron transport package for dose computation. It employs analog photon simulation and a class II condensed history algorithm for electrons. Soon after, it was extended to support electron transport in a magnetic field (Hissoiny *et al* 2011b). In 2019, a proton dose computation version, pGPUMCD (Maneval *et al* 2019), was introduced using history-based proton transport.

In Geant4-based developments, two packages emerged from different research groups. GMC (Jahnke *et al* 2012) was introduced in 2012, using an event-based approach for coupled photon–electron transport and dose computations in voxelized geometry. Meanwhile, GGEMS (Bert *et al* 2013) was developed in 2013 based on a history-based method for coupled photon–electron transport. In 2015, GGEMS was extended for low-dose-rate brachytherapy as GGEMS-Brachy (Lemaréchal *et al* 2015), incorporating analytical geometry for radiation seed modeling.

Recently, GPU acceleration of coupled photon, electron/positron, and neutron transport for medical applications was implemented in the RT2 package (Lee and Ye 2024). This package utilizes RT cores for accelerated computation, employing triangle-meshed geometry and an event-based transport strategy. For dose calculation applications, RT2 demonstrated speedups of 150–300 times for photon and electron transport, and up to 135 times for neutron transport, compared to FLUKA.

#### 4.3.2. Image simulation engines

PENELOPE-based MCGPU (Badal and Badano 2009) was developed in 2009 for CBCT and mammography applications, employing history-based photon transport in voxelized geometry. The code was upgraded in 2021 with a more realistic image formation model and a binary tree-based geometric model for handling extremely large-resolution voxelized phantoms, and used to replicate a commercial mammography and breast tomosynthesis imaging device in a virtual imaging trial (Badal *et al* 2021). In 2024, it was expanded to support PET simulation as MCGPU-PET (Herraiz *et al* 2024), employing a simplified ideal detector without optical light transport. Separately, another group built UMC-PET (Galve *et al* 2024) in 2024, also using PENELOPE as the underlying engine. In UMC-PET, both the phantom and the PET detector are modeled with voxelized geometry, resulting in a 1500–2000-fold speedup compared to PeneloPET.

EGS-based CUDA EGS (Lippuner and Elbakri 2011) was introduced in 2011, using history-based photon transport in a voxelized geometry for diagnostic image simulation. An ideal detector was assumed.

Building on the gCTD architecture (Jia *et al* 2012c), gDRR was developed in 2012 (Jia *et al* 2012b) to incorporate simulations of bowtie filters, polyenergetic spectra, and detector responses for realistic CBCT projection imaging. Using the parameterized geometry expansion from gDPM (Chi *et al* 2016), the same group introduced gPET (Lai *et al* 2019) in 2019, a GPU-based PET simulation platform that supports positron decay and range estimation, gamma transport within voxelized phantoms and parameterized scintillation detectors, as well as digitizing process.

Geant4-based GGEMS (Bert *et al* 2013) provides not only dose computation but also emission and transmission image simulation. For transmission imaging, it uses a flat-panel detector, while for emission, gammas exiting the phantom are recorded in a phase-space file. In 2016, it was extended to GGEMS-SPECT (Garcia *et al* 2016), incorporating analytical geometry for the SPECT head camera modeling. GPU-based MC covers the entire process from gamma generation in voxelized phantom to pulse generation in the analytical SPECT camera. In 2019, it was further extended to support PET simulation as GGEMS-PET (Ma *et al* 2019). In this package, GPU-based MC simulation covers the process from gamma generation in voxelized phantom to its detection in voxelized scintillation detectors.

In the field of optical tomography, several GPU-based MC codes have also been developed. GPU-WMC (Alerstam *et al* 2008), introduced in 2008, was an early implementation designed for homogeneous tissue, and achieved over 1000-fold speedup compared to a single CPU. GPU support for photon migration in voxelized turbid media was introduced in 2009 with MCX (Fang and Boas 2009) and further advanced in 2010 with MCML-GPU (Alerstam *et al* 2010), both achieving several hundred-fold speedups over CPU implementations. Additional efforts have focused on supporting photon transport in more realistic tissue geometries using meshed or hybrid voxel-mesh representations. Early implementations relied on constant and texture memory to perform ray-triangle intersections, with modest speedups under 10-fold (Ren *et al* 2010). More recent advances have dramatically improved efficiency, achieving speedups of several hundred-fold using advanced ray tracing algorithms and hardware-accelerated RT cores (Fang and Yan 2019, Yan and Fang 2020, Yan 2024).

In the field of ultrasound tomography, MCPT (Mattausch *et al* 2018) was developed in 2018. It introduced interactive MC path tracing for more realistic modeling of ultrasound transport over complex surfaces using both RT and CUDA cores. Building on the success of MCPT, several research groups have developed other GPU-based MC path tracing methods for ultrasound imaging. For example, CUS (Amadou *et al* 2024) was developed in 2024 for cardiac ultrasound imaging, featuring segmentation and voxel-based geometry rather than mesh-based modeling for ray tracing. UltraRay (Duelmer *et al* 2025) further extended MC-based path tracing into a full-path simulation from emitter to receiver. It also provides versatile support for different echo acquisition modes and an end-to-end ultrasound image formation.

### 4.4. Applications

GPU-based MC simulation packages have significantly contributed to the design and evaluation of novel imaging systems. To name just a few among many applications: they have been used for scatter estimation in multi-source or photon-counting CT (Sharma *et al* 2019), spectral optimization in advanced CT systems (Wang *et al* 2024), organ-specific dose estimation (Wang *et al* 2025), comprehensive CBCT dose estimation (Montanari *et al* 2014), scatter correction in CBCT (Zhang *et al* 2020), sensitivity and resolution estimation for new PET system designs (Lai *et al* 2021), scatter correction in brain PET (Noë *et al* 2024) and brain PET/MRI (Ma *et al* 2019), image reconstruction for SPECT/CT system (Rydén *et al* 2018), pushing boundaries in TOF and quantitative PET imaging (Lecoq *et al* 2020, Meikle *et al* 2021), among others.

Over the past decade, AI has seen widespread application in advancing medical imaging technologies (Pesapane *et al* 2018, Barragán-Montero *et al* 2021). A critical factor in reliably training AI models is the availability of high-quality, physically accurate data. GPU-based MC simulations have played an essential role in generating such synthetic data with a high efficiency (Sizikova *et al* 2024), supporting tasks including patient-specific dose estimation (Roser *et al* 2019b, Morató *et al* 2025), CT/CBCT scatter estimation (Zhuo *et al* 2023, Agrawal *et al* 2024a, 2024b, Xia *et al* 2025), virtual image generation (Madesta *et al* 2024), among others (Sarrut *et al* 2021).

Most recently, the concepts of digital twin for health (DT4H) (Katsoulakis *et al* 2024) and virtual clinical trials (VCTs) (Abadi *et al* 2020b, Samei *et al* 2025) have drawn considerable research interest in the field of medical imaging. DT4H refers to a virtual representation of an individual, enabling dynamic simulation of potential treatment strategies, monitoring and predicting health trajectories, and facilitating early intervention and prevention. This is achieved through multi-scale modeling of multimodal data, including clinical, genetic, molecular, environmental, and social factors. VCTs, on the other hand, simulate patients, imaging systems, and interpreters to virtually evaluate medical imaging technologies. GPU-based MC simulation is particularly well-suited for these applications, as it offers both reliability and computational efficiency in performing the required simulations, while avoiding the explainability and hallucination issues found in machine-learning-based image generation models (Roser *et al* 2019a, Abadi *et al* 2020a, 2024, Badal *et al* 2021, Mettivier *et al* 2022).

## 5. Discussion and future directions

As we have shown, GPU techniques have enabled the development of various high-performance MC simulation packages for tomographic applications, often achieving speedups of two to three orders of magnitude over CPU implementations. These MC platforms have provided essential support in the development of novel imaging systems with improved image qualities. To further support emerging research needs and new imaging concepts, such as novel imaging principles, hybrid modalities, ML, DT4H, and VCTs, GPU-based MC simulation is expected to continue playing a critical role, potentially in an evolved form. On the one hand, GPU-based MC simulation will remain the gold standard to support the development of new technologies and concepts thanks to its capability to accurately model complex physical interactions. On the other hand, developing novel imaging concepts and hybrid modalities demands physics-accurate simulation frameworks capable of adapting to new imaging principles and system designs. Additionally, training and validating ML models require large-scale, realistic synthetic datasets. In DT4H and VCTs, physics-driven and data-driven approaches are anticipated to play equally important roles. To better meet these emerging demands, future GPU-based MC frameworks may need to incorporate new imaging principles, enable flexible and customizable modeling, prioritize high-throughput simulation capabilities, and support efficient uncertainty quantification. Furthermore, evolving GPU-based MC codes from standalone computational tools into tightly integrated, modular components within corresponding imaging ecosystems may also be essential to fully support these emerging innovations.

Technically, a few aspects can be further improved. First, as it is unfortunately common in computer modeling applications, there remains a need to expand the use of standardized methods to report the simulation parameters (Sechopoulos *et al* 2018) and to benchmark the performance of competing simulation codes (Sechopoulos *et al* 2015).

Second, when comparing to CPU-based MC simulations, we should also be aware of some inherent limitations and simplifications with GPU-implementations that might complicate a direct fair comparison. For instance, although single-precision floating-point arithmetic has been widely adopted in medical GPU-based MC simulations and generally does not introduce noticeable artifacts in applications such as dose calculation (Jia *et al* 2010, Magnoux *et al* 2015), it can lead to noticeable rounding errors or precision loss in high-accuracy scenarios, especially when the code is compiled with ‘fast math’ options that prioritize performance over strict IEEE 754 floating-point compliance. As an example, the MCGPU code uses double precision in some key parts of the interaction cross-section sampling and direction rotations to prevent an unrealistic reduction in intensity at certain discrete angles in x-ray projection with very small pixels and large source-to-detector distances. Another limitation is that scientific libraries widely used in CPU programming, such as python’s numpy, are not directly callable from a GPU kernel, requiring the use of simplified GPU-friendly alternatives. As an example of this, GPU-based pseudo random number generators, such as those provided in cuRAND, may not offer sufficient statistical robustness for all use cases, with known failure to certain statistical tests in the TestU01 suite (Manssen *et al* 2012). These generators also have shorter periods than CPU-based implementations, which could cause systematic errors (Hellekalek 1998, Barbone *et al* 2024).

Third, great efforts have been made over the past two decades to accelerate optical photon transport in biological tissues with GPU-based MC techniques (Alerstam *et al* 2008, Zhu and Liu 2013), yet comparable progress with GPU has not been observed for optical photon transport in radiation detectors. This gap is primarily due to the fundamental differences between the two domains. Optical MC simulation in biological tissues primarily involves scattering and absorption processes in heterogeneous media, whereas scintillation detectors utilize transparent materials with little to none bulk scattering and absorption. This means detector performance is largely driven by surface and boundary interactions, making accurate crystal surface characterization critically important (Roncali and Cherry 2013). As a result, accelerating optical MC simulation in radiation detectors, such as in PET detectors, is still in its early stage but is rapidly progressing. Recent efforts have focused on generative AI methods such as generative adversarial networks (GANs) to bypass individual tracking of optical photons in scintillators, an approach now available in the latest GATE version (Trigila *et al* 2023). To accelerate the GAN training, Srikanth *et al* implemented the GAN (called optiGAN) on GPU, obtaining a 4-fold speed gain (Srikanth *et al* 2024).

Fourth, although MC simulation has been found to play important role in supporting pCT development, such as for image reconstruction, RSP estimation, and detector effect study (Dedes *et al* 2022) and there have been various GPU-based MC simulation code developed to support proton radiotherapy (Jia *et al* 2012a, Wan Chan Tseung *et al* 2015, Schiavi *et al* 2017, Luo *et al* 2025), including those for proton in-beam PET simulation (McNamara *et al* 2022), there has been no dedicated GPU-based MC package developed for pCT to the best of the authors’ knowledge. Researchers often need to rely on CPU-based general purpose MC simulation packages, such as Geant4 and TOPAS for pCT studies (Dedes *et al* 2022). Along with the fast development of pCT and its potential application in radiation clinic in near future, GPU-accelerated MC simulation packages that support both dose computation and image simulation are likely to become increasingly important.

Fifth, it is noteworthy from table 2 that most existing GPU-based MC simulation codes for emission and transmission tomography applications utilize a history-based transport strategy. This approach is generally more GPU programmer friendly and offers advantages for certain types of simulation analysis, such as track structure modeling and radiation dose uncertainty estimation (Uehara *et al* 2001, Walters *et al* 2002, Chetty *et al* 2007). However, along with the incremental performance of GPU cards, event-based transport may have a larger efficiency improvement space. Hamilton and Evans (2019) compared history-based and event-based algorithms for a GPU-based continuous-energy MC neutron transport solver and reported interesting findings. They found that event-based algorithms significantly outperformed history-based approaches, which is even more obvious with the latest generation of GPUs (see figures 9 and 10 in Hamilton *et al*’s work (2019)). On the other hand, if adhering to history-based approach, applying new algorithms to further reduce thread divergence can be an option. For instance, various efforts in non-medical MC applications have been made to develop SIMT-friendly random sampling techniques aimed at improving efficiency (Biondo *et al* 2021, Ridley *et al* 2024). These methods show promising potential for adaptation and application in the medical domain.

Sixth, with the advancement of GPU hardware, such as hardware-accelerated RT cores and highly efficient tensor cores, a shift from traditional CUDA core-focused acceleration (with custom made geometrical models) toward taking advantage of these specialized cores offers new opportunities to further boost MC simulation performance. Recent applications of RT cores to accelerate MC simulations in both medical and non-medical fields (Salmon and McIntosh-Smith 2019, Lee and Ye 2024) have demonstrated this potential, inspiring further efforts to fully exploit these hardware capabilities. Meanwhile, although tensor cores were originally designed to accelerate matrix operations, some researchers in non-tomography fields have begun to explore their potential to accelerate MC simulations, achieving promising performance gains over CUDA core-only implementations (Aitchison 2019, Yang *et al* 2019). These findings suggest potential applications in tomography, particularly for MC models that can be reformulated to exploit dense matrix operations or batched transport strategies.

Last but not the least, it is also worth noting that although the application of GPUs has significantly boosted the performance of MC simulations, GPU code portability remains an area that deserves further exploration. Within the proprietary NVIDIA CUDA ecosystem, although a given CUDA version is generally compatible with GPUs of different computational capabilities, variations in hardware execution can cause discrepancies in computational results, even when using the same high-level code. This necessitates careful examination of CUDA version and computing capability changes to ensure consistency and maintain code integrity. Hardware changes limit the repeatability of published studies, and might even make existing open-source codes unusable in future GPUs (contrasting with the ease of compiling ancient CPU-based FORTRAN software). Meanwhile, upgrading to newer CUDA versions often requires manual code modifications, and in some cases, it may require major code revisions to fully exploit hardware advancements. Also due to the proprietary nature of CUDA, porting CUDA code to other platforms, such as AMD GPUs, typically demands additional effort, although there exist tools like HIPIFY to assist with migration. Cross-platform models like OpenCL and OpenMP offer greater flexibility but may face limitations in fully accessing platform-specific hardware capabilities, potentially restricting performance. Overall, to maximize the role of GPU-based MC simulations in advancing tomography development, further improvements in both hardware and software portability support will be essential.

## Data Availability

All data that support the findings of this study are included within the article (and any supplementary information files).
